# Mapping the Clinical Care Pathway of Fragility Fracture Patients at a German Maximum Care Provider Through Qualitative Research

**DOI:** 10.1055/a-2658-0326

**Published:** 2025-08-08

**Authors:** Gábor Köhalmi, Patrick Dirks, Dorian Fass, Romy Bley, Hans Derk Pannen, Ralf Kuhlen, Andreas Bollmann, Nina Voigt, Clayton Kraft

**Affiliations:** 1 Helios Health Institute, Berlin, Germany; 2 14050 RWE, UCB Pharma GmbH, Monheim am Rhein, Germany; 3 14050 Medical Strategy, UCB Pharma GmbH, Monheim am Rhein, Germany; 4 27664 Clinic for Orthopedics, Trauma- and Hand Surgery, HELIOS Klinikum Krefeld, Krefeld, Germany

**Keywords:** osteoporosis, fragility fractures, clinical care pathway, qualitative research, pathway, Osteoporose, klinischer Behandlungspfad, qualitative Forschung, Ffragilitätsfraktur, Behandlungspfad

## Abstract

Osteoporosis is a chronic underdiagnosed condition that weakens bone structure with increased risk of fragility fractures. While the prevalence of osteoporosis is expected to increase due to demographic developments in many countries, there is found to be a serious treatment gap for patients. This is partly due to inadequate diagnostic procedures at healthcare facilities. Considering this, there is a need to understand factors that affect processes involving diagnosis and treatment in osteoporotic patients. This study’s primary aim is to explore the management of patients with fragility fractures and osteoporosis by conducting and analyzing semi-structured interviews with healthcare professionals at a German maximum care provider. Insights from the interviews were used to map out the pathway of clinical care for patients and the results suggest a multitude of factors including disease awareness, communication, and up-to-date information to be particularly important for increased treatment quality. Future studies shall focus on improving generalizability and exploring the effectiveness of recently updated guidelines for management of osteoporosis.

## Zusammenfassung

Osteoporose ist eine chronische, unterdiagnostizierte Erkrankung, die die Knochenstruktur schwächt und das Risiko für Fragilitätsfrakturen erhöht. Aufgrund der demografischen Entwicklungen in vielen Ländern wird eine Zunahme der Prävalenz von Osteoporose vermutet, jedoch besteht eine erhebliche Behandlungslücke bei Frakturpatienten. Dies ist teilweise auf unzureichende diagnostische Verfahren in Gesundheitseinrichtungen zurückzuführen. Daher ist es notwendig, die Faktoren zu verstehen, welche die Prozesse der Diagnostik und Behandlung von Osteoporosepatienten beeinflussen. Ziel dieser Studie ist es, das Management von Patienten mit Fragilitätsfrakturen und Osteoporose zu untersuchen, indem semistrukturierte Interviews mit medizinischem Fachpersonal eines deutschen Maximalversorgers durchgeführt und analysiert werden. Die Erkenntnisse aus den Interviews wurden genutzt, um den Behandlungspfad klinisch abzubilden. Die Ergebnisse deuten darauf hin, dass eine Vielzahl von Faktoren, einschl. Krankheitsbewusstsein, Kommunikation und aktueller Informationen, für eine erhöhte Behandlungsqualität besonders wichtig sind.

## List of Abbreviations

DRG: Diagnosis-related group
DVO: Dachverband Osteologie e. V.
DXA: Dual-energy X-ray absorptiometry
ER: Emergency room
FLS: Fracture liaison service
GP: General practitioner
MOF: Major osteoporotic fracture

## Background

Osteoporosis is a chronic condition characterized by reduced bone mass and deteriorated bone structure resulting in increased bone fragility and propensity for fractures. Findings from the “Broken bones, broken lives” report by the International Osteoporosis Foundation indicate that the aging population will lead to an increasing number of fragility fractures in the coming years, resulting in significant clinical, societal, and financial burden in Germany 1 .

Osteoporotic fragility fractures can have a significant impact on patients’ quality of life, leading to increased morbidity and mortality. In 2019, Germany had more than 830,000 cases of fragility fractures, and crude incidence of such fractures stood at 22.2/1,000 in the population aged 50 and above 2 . The most prevalent fragility fractures are fractures of the spine (vertebrae), hip (femur), and wrist (radius) 3 . Hip fractures are amongst the most prominent indicators for osteoporosis 4 . Differences in types of fractures exist among distinct age groups. Patients between the age of 40 and 60 usually tend to suffer from radius or vertebral fractures 4 , while patients between 60 and 75 years of age have additional exposure to (proximal) humerus fractures 5 . Elderly people over 75 or even over 85 years tend to suffer increasingly with the consequences of hip fractures as well as vertebral and pelvic fractures 6 7 .

An estimated 23 million individuals in the European Union are affected by osteoporosis, making it a significant public health concern 2 . This is especially true for women. About one-tenth of women aged 60 years, one-fifth of women aged 70, two-fifths of women aged 80, and two-thirds of women aged 90 face increased risk of osteoporosis and fragility fractures 8 . While precise prevalence rates for osteoporosis have been cited as difficult to estimate due to the condition’s silent nature, lifetime risk of sustaining fractures is expected to increase in countries with ageing populations 9 . Therefore, enhanced fragility fracture treatment, post-fracture care, and early diagnosis of underlying diseases and risk factors will become increasingly important for healthcare providers. A particularly significant treatment gap of 76% for osteoporosis patients has been identified in Germany with lack of adequate osteoporosis diagnosis for patients at increased risk of fragility fractures as a major cause 10 .

The negative consequences of fragility fractures on patient health and quality of life make it crucial to further evaluate and improve diagnosis and treatment of osteoporosis. While research exists on improving osteoporosis care 11 , there is a lack of specific studies on exploring barriers and challenges from the hospital perspective to complete the picture. Existing research in the German context focuses on quantitative aspects of potential implemented measures without considering the perspective of hospital staff 12 . This implies that the scientific community is aware of both the high prevalence of fragility fractures and the existing treatment gap for affected patients. Obtaining a better understanding of the underlying causes for the current care situation requires additional qualitative insights by collecting data from those working directly with the relevant patient groups. Therefore, this qualitative study is aimed towards understanding clinical care as it stands and its challenges in the management of fragility fracture cases by conducting semi-structured interviews with healthcare professionals across various areas at a German maximum care hospital. By exploring their various perspectives, this study sought to further analyze the journey of fracture patients in order to identify potential areas for improvement along the clinical treatment pathway.

## Methods

### Choice of Methods

Qualitative methods can generally yield in significant insights even from a low number of respondents. This is therefore an effective method to understand complex situations.

An exploratory and foundational research approach was required to provide an overview of relevant clinical processes, given the current lack of understanding of the clinical pathway in patients with fragility fractures. Consequently, semi-structured interviews with clinical experts, pathway visualization, and validation of the key findings in a workshop were identified as key methods to gain a comprehensive understanding of clinical habits and procedures. The chosen approach captured the full spectrum of variables defining the treatment pathway, establishing a basis for effective decisions for improvement and follow-up research. The aim of this study was to explore the entire hospital treatment path starting with admission and continuing along diagnosis, fracture treatment, and inpatient stay, concluding with discharge and aftercare.

### Endpoints

The primary endpoint of this study was to understand the central elements and patterns of the treatment of patients with fragility fractures in Germany, including the identification of treatment challenges faced by healthcare professionals.

As a secondary endpoint, this study intended to validate initial findings from additional physicians from other hospitals to include additional perspectives on treatment realities. Another secondary endpoint aimed at deriving pragmatic suggestions for decision-makers to improve quality of care in the future.

### Semi-structured Interviews

**Fig. 1:**
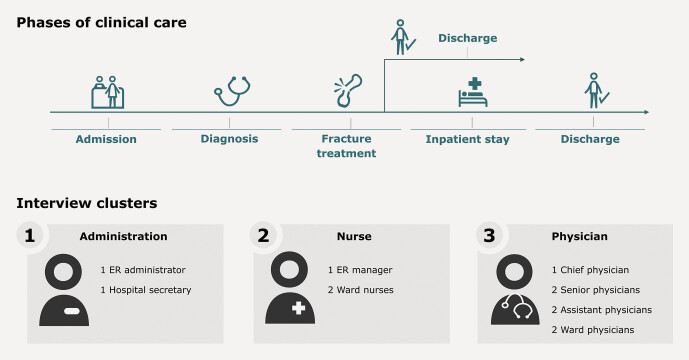
Schematic overview of the care structure as well as the clusters for each interview group. Source: Helios Health Institute GmbH, Berlin.

In order to refine the hypothesis and finalize the questionnaire, the medical project leads surveyed the consensus on the current state of clinical care for patients with fragility fractures in Germany. Given the fact that potentially osteoporotic patients primarily arrive at the hospital via the ER due to acute fractures (most frequently humerus, radius, vertebrae, or femur fractures), these “major osteoporotic fractures” (MOFs) were considered as the main fractures of interest when developing the interview questionnaires (see Appendix 1). The interview structure was defined together with healthcare professionals describing the major phases of clinical care from admission through diagnosis, fracture treatment, inpatient stay, and discharge. This enabled the researchers to gather their hypotheses and core questions for the exploration phase. Twelve healthcare professionals were selected for the interviews at a German hospital with maximum care; these included physicians, nurses, and administration staff. The selection was primarily based on two factors, a) experience with treating patients and b) the variety of functions and departments. An overview of the interviewees can be found in Fig. 1 .

Interviewees were selected according to the following criteria:

- Employed at Helios Klinikum Krefeld
- Aged more than 18 years old
- Written consent for participation
- Current experience with/exposure to patients with fragility fractures

**Fig. 2:**
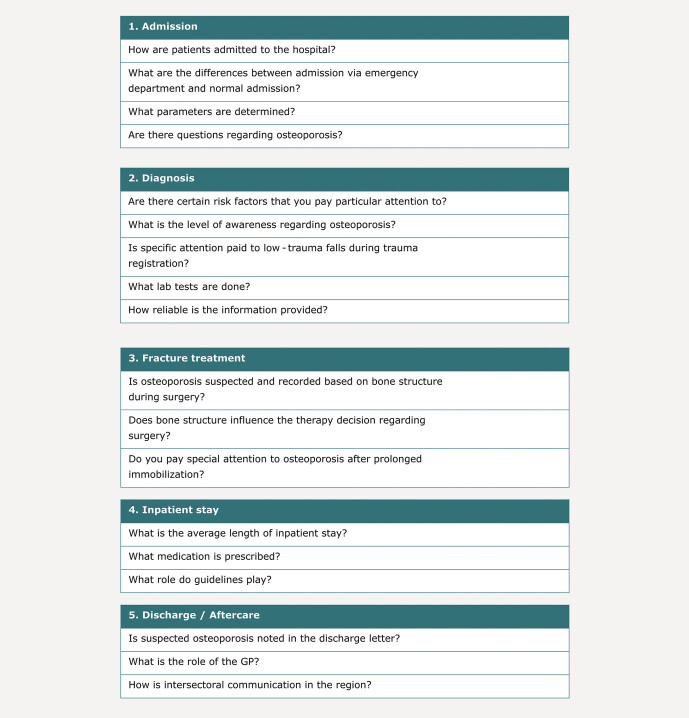
Key themes for the interview script organized in the five phases of clinical care.

To ensure comparability of the interviews, a script with guiding questions was developed addressing the most relevant aspects of fragility fracture treatment and diagnosis ( Fig. 2 ). Core questions for each phase (admission, diagnosis, fracture treatment, inpatient stay, discharge/aftercare) focused on exploring the interviewees’ experience with additional in-depth questions on pain points and areas of improvement, where possible.

Each interview spanned over 90 minutes and was conducted by a trained interviewer and a note taker, ensuring adherence to the script while allowing open-ended responses at the same time. At the beginning of each interview, the phases of the clinical care structure were presented to the interviewees in card format for a visual overview. Between the interviews, results were reviewed and discussed by the project team and the answers were completed with observations or additional information if required.

After all twelve interviews had been conducted, the notes for each interview were reviewed again according to the predefined structure. Finally, key insights were mapped out and organized along the clinical pathway creating a preliminary version, displaying the current state of clinical care of patients with fragility fractures.

### Clinical pathway

The interview results were processed using the Mayring method 13 and followed the phases of preparation, coding and concluding. A visualized clinical pathway – a diagram showing patient steps in chronological order from left to right – was created following the conclusion phase. This allows readers not just to acquire a birds-eye view of the whole clinical treatment process, but also to zoom in on specific steps and understand respective problems and aspects in that specific area.

In the preparation phase, the interview results were clustered into three separate groups to reflect the perspectives from physicians, nurses, and administrators for use in the final visualized patient pathway. In the coding phase, the most relevant insights were organized along the core pathway structure. In the concluding phase following revisions, a final structure was developed to illustrate how patients navigate clinical care for fragility MOFs displaying interactions between patients and care providers and highlighting events of importance, pain points, and notable observations.

### Workshop

The pathway was presented to an expert panel to evaluate the patient pathway and discuss and identify potential improvement options for enhanced clinical management. This panel consisted of a chief trauma surgeon and orthopedic specialists from two additional hospitals from the same hospital group. An assessment was made as to whether the insights collected could be considered representative of the clinical processes and care provided in hospitals of different sizes. Following the validation, a series of steps were conducted to reach consensus within the expert panel on key weaknesses, gaps, and redundancies in the current state of care:

First, the pathway visualization was presented to the experts, followed by an exercise referred to as silence commenting. This step was for participants to collect questions, comments, and corrections on sticky notes and attach them to the respective part of the pathway. Second, the comments were reorganized and read out loud to the participants by the moderator, each followed by a time-restricted group reflection and discussion. Third, based on the discussions, the participants were asked to mark areas on the pathway that present opportunities to improve either clinical care for fragility fractures and/or the patient experience along the delivery of care. The workshop ended with a discussion on possible improvement options and opportunity areas based on the results. These opportunity areas included touchpoints and process steps where developing new solutions could lead to added value for the patients with fragility fractures and enhanced management of osteoporosis. All results and findings from this expert workshop were implemented in the final version of the patient pathway.

## Results

**Fig. 3:**
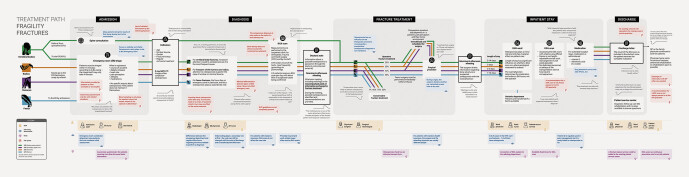
Overview of the treatment path of fragility fracture patients. Source: Helios Health Institute GmbH, Berlin.

Interviews, the workshop, and the visualized clinical pathway revealed a range of pain points and observations across the entire treatment process. Fig. 3 shows an overview of the treatment pathway. The original document can be found in Appendix 2.

### Admission

Femur fractures are entirely diagnosed in the ER, but all other MOFs were diagnosed at general admission and in the emergency room in equal proportions. Unless patients specifically mention underlying conditions or are evidently suffering from low-impact trauma on admission, medical staff will primarily address the most urgent and severe condition without considering underlying risk factors or circumstances such as osteoporosis. This particularly applies to the emergency room, as time is usually scarce compared to general consultations.

Patients occasionally bring results of bone density tests, but information provided by patients or their referring physician generally appears to be unreliably subjective or incomplete overall.

### Diagnosis

Results regarding diagnosis can be organized in two main categories: information and diagnostics.

**1. Information**

Physicians and nurses receive the most relevant patient information in the form of doctor’s notes and daily department meetings. Initial diagnostic coding plays a secondary role here since codes are subject to change until the patient has been discharged. As noted by interviewees, these meetings and ward rounds are the most important points of information exchange between teams, for example to communicate suspicion of osteoporosis.

**2. Diagnostics**

Dual energy X-ray absorptiometry (DXA) scans are seen as the current gold standard for osteoporosis diagnosis. Nonetheless, high barriers against reimbursement for this non-invasive and effective diagnostic instrument from the German statutory insurance system proves to be a major limitation to according to interviewees. Therefore, x-ray examinations and much needed DXA scans are rarely performed at the same time. It was further noted that the current guidelines issued by the Dachverband Osteologie (DVO) at the time the interviews were conducted were out of date and needed adaptation to adequately guide physicians towards osteoporosis as a diagnosis.

### Fracture treatment

Theoretically, there is an opportunity to diagnose osteoporosis prior to surgery using DXA scans in many cases. Even though this may well change the extent and type of surgery performed (e.g. use of additive bone cement or specific implants), little use is made of this potentially valuable information since osteoporosis has little to no influence on the therapy as reported by interviewees. However, diminished bone density is frequently encountered during operative fracture care and bone fixation. Consequentially, surgeons often need to adjust the procedure in the operating theater while performing surgery. Despite the mechanical proof that bone structure is insufficient, this important information is often not passed on to subsequent care providers. In contrast, awareness for osteoporosis in conservative fracture management without surgery was reported to be enhanced as the bone structure is pivotal to a successful healing process. Interviewees estimated that on average, about 60% of MOFs are treated through conservative care except femur fractures, which require expedited surgical treatment within 24 hours after admission.

### Inpatient stay

The length of stay differs among MOFs, but osteoporosis only plays a minor role for ward physicians responsible for the therapy until discharge, focusing on soft tissue and bone healing as well as mobilization. However, ward physicians play a key role in deciding whether to perform DXA scans or pharmacological treatment. All ward physicians and nurses interviewed mentioned that length of hospital stay usually correlates with increased rates of suspected or diagnosed osteoporosis due to additional examinations and more frequent patient-physician encounters. All interviewed ward physicians noted that in many cases, recommendations for DXA scans are not passed on to general practitioners (GP) or other specialists in the outpatient sector, providing further care, and potentially resulting in more cases of suspected osteoporosis than actual diagnoses. Within the hospital, DXA scans deemed necessary appear to be rarely conducted due to difficult physical access, lack of personnel, and time constraints, as the device is not located in the radiology department. Moreover, all interviewed ward physicians confirmed that the scan is not necessary for immediate healing, which explains the tendency to suggest but not perform these scans.

### Discharge

The discharge letter is usually the most common method of sharing information between hospital doctors and the GP or outpatient specialist providing post-clinical care. If conducted, the complete evaluation including DXA scan results are included in the letter. In clinical routine, however, discharge letters are usually written under tight time constraints, and the focus lies on the primary fracture condition rather than potential underlying conditions such as osteoporosis. As a result, even if suspected during the hospital stay, this vital information might not be documented and shared. All interviewed ward physicians have confirmed that patients are not directly referred to an osteologist regardless of diagnosis. This is mainly due to a lack of network structures between hospitals and outpatient specialists. In general, there appears to be little direct communication between the hospital and GP before and after inpatient care.

### Pain points

**Fig. 4:**
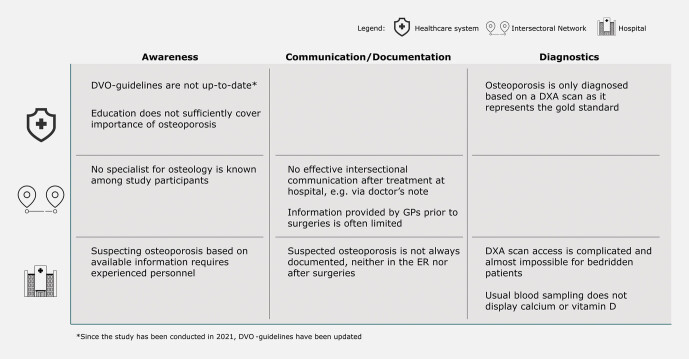
Summary of identified pain points categorized by healthcare system, intersectoral network, and hospital-level. Source: Helios Health Institute GmbH, Berlin.

As part of pathway development, the project team identified the most relevant challenges in clinical care for fragility fractures and framed them as pain points. The most striking ones were attributed to communication/documentation, diagnostics, or awareness ( Fig. 4 ). These pain points most prominently hamper successful early identification of osteoporosis regardless of factors influencing any type of diagnosis such as time constrains, limited information provided by the patient, or focusing on the primary/most severe condition – in this case the fractured bone.

There is room for improvement in the communication between outpatient care providers and hospitals in view of the strict differentiation between inpatient and outpatient treatment as two fundamental pillars of the German healthcare system. This is considered to be a general issue for a variety of indications and diseases but applies especially to osteoporosis as a notoriously underdiagnosed disease. This study also highlighted the shortage or absence of information provided by GPs prior to surgical treatment. On the other hand, this also holds true if osteoporosis is suspected within the course of a hospital stay. Information is often not transferred to the GP or specific outpatient specialist, who might then fail to initiate or continue the required post fracture care. Only a minority of study participants were able to name or recommend an expert or excellence center specialized in osteoporosis care for outpatients.

The issue of inadequate communication correlates not only with awareness of osteoporosis but also with the experience of physicians. Suspecting and deriving indications for low-trauma fractures based on medical history, course of events, and lifestyle described orally by patients requires experienced personnel. Nevertheless, it is worth mentioning that all interview participants were well aware of the negative consequences of osteoporosis on patients’ health and quality of life and agreed that these issues should generally be addressed by GPs in outpatient care.

An additional main pain point is documentation and thus communication between different clinical departments once osteoporosis is suspected at any stage in the patient pathway. Medical staff often do not document or communicate suspected diagnoses not primarily attributed to the main diagnosis. The physician in the emergency room (ER), treating physician or surgeon, and ward physician are most likely different individuals, so quick information exchange in the form of documentation is of essential importance. Doctor’s notes, medical reports, handovers, medical ward rounds, and morning and afternoon meetings are effective means for such exchange.

The third pain point involves diagnostics. According to interviewed participants, osteoporosis is usually diagnosed only after a DXA scan has been performed to confirm the diagnosis. However, access to DXA scans is not only complicated but close to impossible for bedridden patients due to limited space in scanning rooms. Additional diagnostic parameters that could indicate lower bone density such as calcium and vitamin D are not part of a standard blood laboratory workup as they play a minor role in immediate trauma treatment.

### Opportunities for care improvement

During the expert workshop, diagnosis and discharge management were identified as the most promising areas for improvement of care along the patient pathway. Osteoporosis could be diagnosed at various patient encounters during the surgical or conventional treatment. In addition, postoperative care at the hospital provides further opportunities to improve treatment quality. Following fracture treatment, ward physicians have an opportunity to clarify the circumstances under which the fracture occurred, especially when dealing with unspecific trauma cases. In conclusion, suspicion and diagnosis of potential osteoporosis should not be limited to initial consultation when treating patients with MOFs.

Discharging the patient, briefing the patient on following steps, and post-fracture care represent the second area of opportunities. In addition to improving the communication and the sharing of information with the referring physician or GP, patient-centered and holistic outpatient treatment support could represent an opportunity in managing patients with osteoporosis. It might therefore be worth considering how potential options could take shape in clinical care for fragility fractures and beyond towards improving management of osteoporosis in future.

To this end, the participants validated the results and generated new ideas with the goal of improving quality of life for the questions were defined, solutions were generated and organized in an impact-to-complexity matrix (also referred to as an *action priority matrix* ) during the expert workshop. This matrix ranks opportunities according to feasibility and serves to differentiate between easily implementable “quick wins” and more complex projects. This is an effective tool to quickly assess the potential of ideas based on their effort and expected impact 14 .

Clinical processes focus on primary care, i.e. fracture treatment in the context of this project. However, structures worth discussing are those that help identify osteoporosis as secondary diagnosis. Therefore, the first two strategic questions discussed during the expert workshop address initial diagnosis of osteoporosis in the clinical setting: How can the diagnosis rate for osteoporosis be increased? How can the awareness of osteoporosis among the medical staff be improved?

**Fig. 5:**
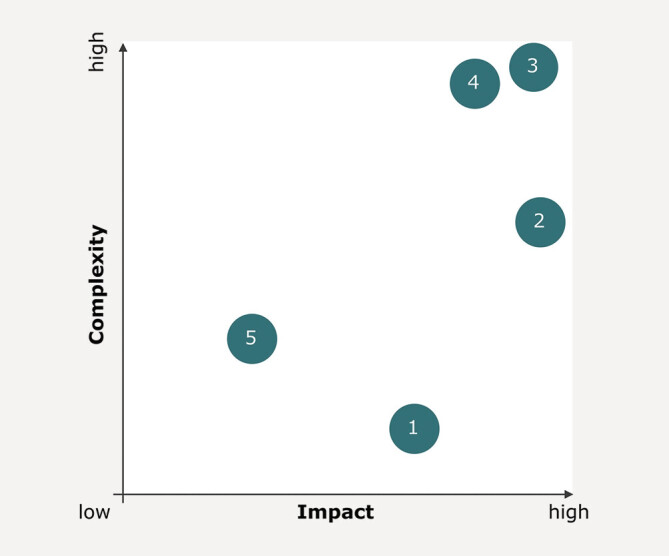
Action priority matrix of solutions for improving diagnosis of osteoporosis in terms of their expert assessment of complexity and impact. The x-axis indicates the degree of impact of a solution on clinical care and ultimately on patients with fragility fractures. The y-axis shows the complexity of implementing the solution.

The following opportunities have been identified and ranked by the participants in the expert workshop ( Fig. 5 ). The individual items are numbered in ascending order in accordance with its impact relative to its complexity. For example, idea number one was given medium impact with low complexity, and idea number three received a potential for high impact but also high complexity. The descriptions corresponding to each number can be found in Table 1 .

**Table:** **Table 1** Description of items from Fig. 5 .

#	Description
1	Physicians’ and patients’ awareness of the disease, the associated costs, and impact on society as well as on individuals need to be raised. More frequent communication via meetings or information flyers would lead to an increase of awareness in clinical routine for physicians. Well-informed patients would be better able to meet physicians at eye level.
2	An algorithm and consequentially a medical device should be established for early diagnoses to specifically target and identify patients at high risk of osteoporosis. Early bone density measurements followed by repeated scans would facilitate both treatment decisions and timely detection.
3	The introduction and establishment of regular medical check-ups could serve as a preventive action. This would cover two important aspects at the same time: On the one hand, this would raise awareness of the disease; on the other, it would support early detection.
4	Guidelines ^1^ need to be reviewed, changed, or expanded to reflect the status quo while emphasizing the importance of osteoporosis as an underlying disease and risk factor for subsequent fractures.
5	Provide care models for patients from diagnosis to surgery to rehabilitation, including all relevant protagonists. Effective network structures are a prerequisite.
^1^ Author’s note: Guidelines have been updated since the study was concluded, and their potential impact is addressed in the discussion section.	

A further key area for improving clinical care of fragility fractures lies in the discharge process. It might be worth addressing specific questions when rethinking the way a patient with a treated fragility fracture leaves the hospital: Which mandatory information needs to be included in the discharge letter for the benefit of the patient? How can an adequate discharge letter be created earlier on, in the treatment process? How can patients be referred to an adequate (follow-up) specialist?

**Fig. 6:**
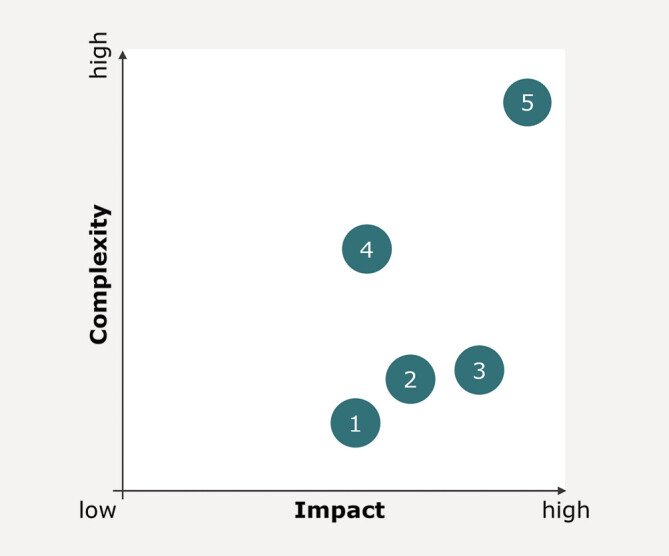
Action Priority Matrix of opportunities for improving discharge processes in terms of their complexity and impact.

The following opportunities have been identified and ranked in the expert workshop ( Fig. 6 ). The order of the numbered options follows the same principle as for Fig. 5 , and descriptions corresponding to each number can be found in Table 2 .

**Table:** **Table 2** Description of items from Fig. 6 .

#	Description
1	Set up an internal network with geriatrics to diagnose osteoporosis more thoroughly and consistently outside of trauma surgery and orthopedics. To make sure that aftercare is organized accordingly, a fracture liaison service (FLS) should be established and connected to the already existing patient service center to provide care for patients eligible for rehabilitation. Such schemes have proven their worth in preventing future fractures and reducing care-related costs 1 .
2	Create a preliminary surgery protocol registering suspected osteoporosis through checklist. This could be used both as a post-surgery information tool for discharge, but also for transfer from ER to ward.
3	Offer additional information material regarding osteoporosis targeting not only patients but also family members.
4	Therapy recommendations should be addressed during initial diagnosis and coded as osteoporotic fracture. While DXA scans are considered the gold standard, efforts should be made to recommend regular bone density tests of any kind, primarily to raise awareness.
5	Expand network structures with physicians in private practices in order to ensure optimized and holistic care (Bund der Osteologen Nordrhein e. V.). Health insurance providers need to be included to support this process.

## Discussion

### Main Findings

In summary, awareness, documentation, communication, and diagnostics play a crucial role in the optimal clinical care of fragility fractures. But it also became clear that in order to improve the current situation, sustainable solutions need to be implemented beyond the clinical setting and should take the intersectoral network as well as wider healthcare system into account. The study conducted suggested that under-diagnosis and thus under-treatment of osteoporosis could be tackled by following the two basic principles, *thinking about osteoporosis* and *communicating and documenting potential suspicions* . In a 2012 study, a survey of over 2000 orthopedic surgeons indicated a lack of knowledge regarding fragility fracture treatment 15 . More recent findings from a 2024 study pointed out the need for increased awareness for fragility fractures 16 , with authors endorsing further improvement in fracture management in accordance with the 2018 Global Call to Action on Fragility Fractures. It is yet to be seen whether awareness for fragility fractures has been improved in the German context.

In order to improve diagnosis rates of osteoporosis, opportunities include increased awareness among patients and physicians but also regularly updating guidelines. This is in line with a recent systematic review in which authors called for increased public awareness and communication for osteoporosis and fragility fractures and also the establishment of osteoporosis as a national health policy to close the gap between demographic challenges and current treatment strategies 17 .

After this study was concluded, the German umbrella association for osteology (DVO) updated their guidelines on September 10, 2023. These guidelines are therefore not part of this study. However, it is important to assess their possible effects on clinical care. These updated guidelines include the extension of the fracture risk score based on the latest scientific evidence with special regard to rheumatism, applying changes to the standard osteoporosis examination for patients with ongoing glucocorticoid therapy, and recommending more individualized osteoporosis drug therapies. Even though the guidelines have been in place for some months, how they will affect routine clinical care and improve early detection remains to be seen.

Finally, efforts are needed for coordinated post-fracture care programs to prevent future fractures; this would include fracture liaison services (FLSs), which have seen increased interest in scientific studies but are still in need of widespread and enhanced implementation in routine care 18 . There is already strong evidence to indicate significant reductions in re-fracture rates for patients at hospitals with an FLS implemented 19 . In Germany, a cluster-randomized, controlled trial has been launched to assess the effectiveness and utility of FLSs and is expected to provide initial results in the coming year 20 . For the time being, public health burden of osteoporosis and fragility fractures remain high.

### Limitations

Due to its nature, this qualitative study has focused on understanding clinical care through semi-structured interviews in a single hospital and can therefore claim limited generalizability. Even though the pathway has been validated by additional physicians from other hospitals, these findings have not been validated outside the hospital group. Moreover, all results have been obtained in the inpatient hospital environment; as such, this study lacks insight on osteoporosis care in the outpatient sector. To produce results on a broader scope, subsequent research should explore additional perspectives by interviewing patients, hospital staff in other geographical areas, as well as ambulatory care providers.

### Conclusion and outlook

Possible reasons for the rather small number of patients diagnosed with osteoporosis in a clinical setting were identified by reference to a clinical care pathway developed at a maximum-care hospital for fragility fractures. A missed diagnosis and an elevated fracture risk due to the first fracture may have a heavy negative impact on patient quality of life in the event of another fracture that could have been prevented. Therefore, it is critical for hospitals to improve their awareness when treating MOFs. One of the biggest obstacles in the clinical environment is the complexity and communication between various clinical structures in determining care processes and responsibilities. A lasting impact would require a multifaceted strategy. One focus should be on strengthening the attention for osteoporosis amongst trauma and emergency physicians who are the first to see patients in the clinical pathway. They play essential roles in defining the course of treatment and therefore have major influence on a possible osteoporosis diagnosis. Once aware, the staff should be able to document or mark osteoporosis with ease in the hospital information system. This should then be supported by up-to-date guidelines that are easily accessible for staff. As a result, osteoporosis will often be appropriately diagnosed in the clinical process and therefore will appear in the discharge letter by default. Physicians responsible for discharge should work together with the patient service center to organize suitable aftercare and establish post-clinical osteoporosis-specific network structures towards improving health and quality of life for post-fracture patients.

## Supplementary Material

Supplementary Material Zusatzmaterial
