# Supplementary material for: Mapping the Clinical Care Pathway of Fragility Fracture Patients at a German Maximum Care Provider Through Qualitative Research
Source: Z Orthop Unfall. 2025 Aug 8;164(1):52–61. doi: 10.1055/a-2658-0326 (PMC12911323; doi:10.1055/a-2658-0326)
Supplement: Supplementary file 1 — Supplementary Material [file 10-1055-a-2658-0326_26581763.pdf]

Appendix 1 – List of questions

ADMISSION

1. What does admission in the ER look like? What is it like for an inpatient admission?
2. How do patients with fractures get to the ER to the hospital? (via ambulance?)
3. What data is recorded here?
4. Who does the patient talk to first?
5. When does the patient see the doctor for the first time?
6. Is blood being taken? If so, what parameters are determined?
7. Are there questions regarding osteoporosis?
8. Is an imaging procedure conducted? If so, which one?

DIAGNOSIS

ANAMNESIS

1. What data is collected by whom and how?
2. How does data get into the HIS?
3. Is there a standard anamnesis form?
4. How are different traumas classified? What is the procedure for unclear traumas?
5. Are there certain risk factors that you pay particular attention to?
6. What procedures are used to aid with the diagnosis?
7. Are old fractures inquired about? Specifically, last year‘s case history?
8. How is coding done and who does it? (M.80)

SUSPECTED OSTEOPOROSIS

9. Are there inquiries regarding osteoporosis or bone diseases, respectively?
10. Do they ask whether a bone density measurement has been performed in advance?
11. What is the level of awareness in trauma surgery among physicians and nursing staff?

12. Is there a structured screening process?
13. Is there a risk questionnaire?
14. What is the role of the DVO guideline?
15. Are there approaches for a "Fracture Liaison Service"? Would this be useful or desired?
16. Is a therapy recommendation already made or even initiated in the hospital?

FALL PROFILE

17. Is specific attention paid to low-trauma falls during trauma registration?
18. Is this information noted?
19. Are fall-inducing medication or medical conditions recorded? If so, how is the information being shared?

LAB

20. What blood tests are done in the hospital?
21. Are small blood counts sufficient for diagnosis? Which parameters are useful?
22. Are bone markers determined? If so, which ones?

DEALING WITH MULTIMORBIDITY

23. What is the role of geriatrics, if existing?
24. What information do you receive from the outpatient department?
25. How reliable is the information provided by patients, relatives and caregivers, and how do you protect yourself in this regard?
26. Do you have access to an existing medication plan?

FRACTURE TREATMENT

SURGERY

1. Is osteoporosis suspected and recorded based on bone structure during surgery?
2. Does bone structure influence the therapy decision regarding surgery? Is osteoporosis being addressed specifically? If so, what are factors, e.g. age?

3. Are treatments recommended by the surgeon? Before or after surgery?

FIXATION

4. Do you pay special attention to osteoporosis after prolonged immobilization?

INPATIENT STAY

ON STATION

1. What is the average length of inpatient stay for osteoporosis patients with fractures*
2. To which ward are fracture patients transferred? Internal medicine/surgery/geriatrics
3. How often does the physician see his patients?
4. What medications do patients receive?
5. When does physiotherapy start?
6. Does rehab already start in the hospital?

SUSPECTED OSTEOPOROSIS (IF APPLICABLE)

7. Are there inquiries regarding osteoporosis or bone diseases, respectively?
8. Do they ask whether a bone density measurement has been performed in advance?
9. Is there a structured screening process?
10. Is there a risk questionnaire?
11. What is the role of the DVO guideline?
12. Are there approaches for a "Fracture Liaison Service"? Would this be useful or desired?
13. Is a therapy recommendation already made or even initiated in the hospital?

DISCHARGE

DISCHARGE MANAGEMENT

1. Is suspected osteoporosis noted in the discharge letter?
2. Who arranges the therapy? Doctor in hospital or the GP?
3. What is the role of the GP?

4. What information do physicians in private practice need?
5. What is the role of rehab facilities in identifiying osteoporosis patients?
6. How are surgical patients in nursing facilities identified and provided with care?
7. Are lab results part of discharge management?

TRANSITION TO OUTPATIENT CARE

8. What role do network structures / patient support programs play when it comes to intersectoral care in the region?
9. How is intersectoral communication in the region?
10. Does digital communication exist?
11. What role do you play in your regional care network?
12. Would you recommend / use patient support programs?

Appendix 2 – Clinical Care Pathway Fragility Fractures

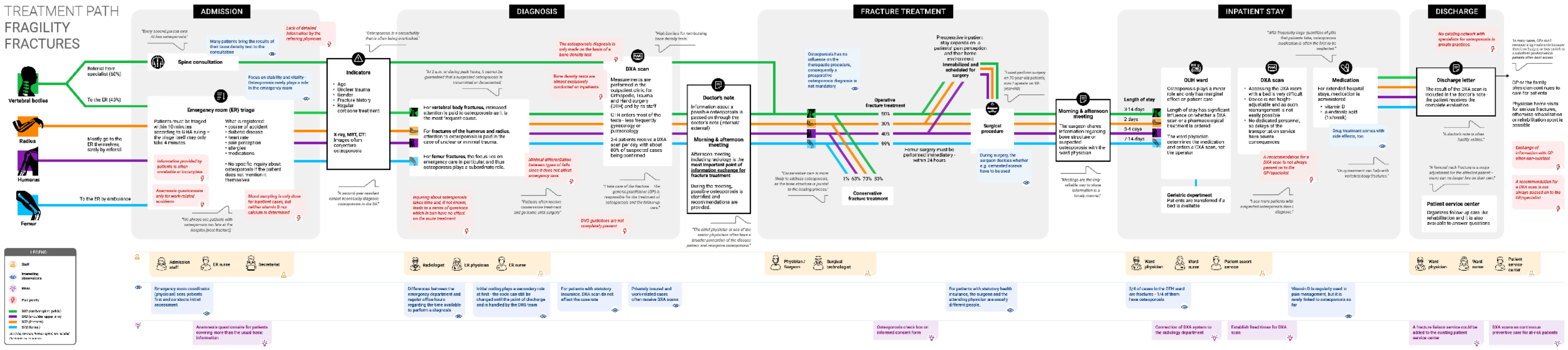

Z Orthop Unfall | DOI 10.1055/a-2658-0326 | © The Author(s).
